# Risk Factors for Persistent or Recurrent Central Serous Chorioretinopathy

**DOI:** 10.1155/2019/5970659

**Published:** 2019-08-14

**Authors:** Jia Yu, Gezhi Xu, Qing Chang, Xiaofeng Ye, Lei Li, Chunhui Jiang, Qi Zhao

**Affiliations:** ^1^Department of Ophthalmology, Eye & Ear Nose Throat Hospital of Fudan University, Shanghai 200031, China; ^2^Shanghai Key Laboratory of Visual Impairment and Restoration, Fudan University, Shanghai 200031, China; ^3^NHC Key Laboratory of Myopia (Fudan University), Shanghai 200031, China; ^4^Laboratory of Myopia, Chinese Academy of Medical Sciences, Shanghai 200031, China; ^5^Department of Epidemiology, School of Public Health, Fudan University, Shanghai 200032, China

## Abstract

**Purpose:**

To investigate the risk factors for persistent or recurrent central serous chorioretinopathy (CSC).

**Materials and Methods:**

Consecutive treatment-naïve CSC patients were included from January 2017 to October 2018. All patients were asked to complete questionnaires, addressing previously described risk factors for the development of CSC. Patients were divided into two groups: those with acute CSC, who were in the first episode, with spontaneous resolution of subretinal fluid within 3 months, and with no recurrence within 1 year; and those with persistent or recurrent CSC, the remaining patients.

**Results:**

In total, 138 patients were enrolled: 20 (14.5%) with acute CSC and 118 (85.5%) with persistent or recurrent CSC. Using multivariate analysis, male sex (odds ratio (OR), 95% confidence interval: 5.63 [1.02–31.02]; *p*=0.047), older age (OR: 1.14 [1.03–1.25]; *p*=0.008), and higher Insomnia Severity Index score (OR: 1.30 [1.05–1.60]; *p*=0.015) were found to be independently associated with persistent or recurrent CSC.

**Conclusions:**

Male sex, age, and sleep disorders are risk factors for persistent or recurrent CSC in the natural history. These patients may require early photodynamic therapy. Treatment for sleep disorders is strongly recommended. All CSC patients may require careful and periodic follow-up.

## 1. Introduction

Central serous chorioretinopathy (CSC) is a common macular disease and often presents with well-circumscribed serous retinal detachment in the macular region on clinical examination, with one or several leakage points at the level of the retinal pigment epithelium (RPE) on fluorescein angiography (FA) [1]. Acute CSC is typically a self-limiting process with few recognized visual sequelae, while chronic CSC and recurrent CSC may develop RPE atrophy and neurosensory retinal changes that result in permanent loss of visual function [2]. Recently, Mrejen has reported that 12.8% of their 133 chronic CSC study patients progressed to bilateral legal blindness [3]. Photodynamic therapy (PDT) has been shown to be effective in promoting the resolution of the subretinal fluid and in reducing recurrence [2, 4–6]. Therefore, it has become a major treatment for CSC, especially for the persistent and recurrent CSC [2, 4–6]. Previous studies have detected several risk factors for the development of CSC, such as male sex, hypertension, depression, allergic disease, stress, smoking, alcohol consumption, shift work, and sleep disturbance [7–12]. However, there are only a few studies on risk factors for persistent or recurrent CSC [13, 14].

The purpose of this case-control study was to investigate the risk factors for persistent or recurrent CSC in a Chinese population using a multivariate approach, thus to help to select the appropriate management strategy for CSC, waiting or PDT.

## 2. Materials and Methods

The approval of the Ethics Committee of Eye and Ear Nose Throat Hospital of Fudan University was obtained, and written informed consent was obtained from all patients before their enrolment in the study. The study adhered to the tenets of the Declaration of Helsinki.

### 2.1. Patients

The participants in this study were consecutive Chinese patients diagnosed with CSC at the clinic of the Eye and Ear Nose Throat Hospital of Fudan University between January 2017 and October 2018.

The clinical diagnosis of CSC was based on symptoms, reduced visual acuity with or without metamorphopsia or micropsia; the presence of serous retinal detachment on both fundus and optical coherence tomography (OCT) examinations; the presence of active angiographic leakage in FA (TRC-50IX; Topcon Corp., Tokyo, Japan); and/or abnormally dilated choroidal vasculature and other features in indocyanine green angiography (Spectralis HRA + OCT; Heidelberg Engineering, Heidelberg, Germany) consistent with the diagnosis of CSC [15].

The subjects included were those who had not been treated with PDT or laser and with clear symptom duration. Excluded were those with other ocular pathologies, such as age-related macular degeneration, diabetic retinopathy, uveitis, optic disc edema, Harada disease, or choroidal infiltrates. Previous studies have identified corticosteroids as a risk factor for the development of CSC, either high levels of exogenous (i.e., intra-articular, intranasal, systemic, or topical) or endogenous (i.e., Cushing's syndrome or pregnancy) [7, 8, 16]. Therefore, we excluded patients using any steroid and those with Cushing's syndrome or pregnancy.

### 2.2. Study Protocol

All patients were asked to complete questionnaires, which included previously described risk factors for the development of CSC [7–12]. Hypertension, depression, allergic disease, tobacco use, and recent psychological stress, including life changes (death, divorce, familial strife, and layoff) and stress at work, were assessed. The abovementioned items were recorded as being either present or absent at the time of presentation. Also recorded were their present and previous year's average monthly alcohol intake, shift work hours per month, sleep beginning time, and sleep hours per day. Alcohol intake was converted to standard drinks, with one standard drink defined as containing 14.0 g of pure alcohol [17], i.e., 350 ml of beer (5% alcohol content), 150 ml of wine (12% alcohol content), or 44 ml of spirits (40% alcohol content). Shift work was defined as work starting before 7 : 00 am or finishing after 7 : 00 pm, and thus evening, night, or early morning work were included within this broader schema. Sleep beginning time was assigned a value, so that 12 midnight was defined as 0, 11 pm as −1, 1 am as 1, and so on. Thus, the sleep beginning time is a continuous variable. All the patients were also asked to complete the Insomnia Severity Index (ISI) questionnaire, which is an instrument widely used to evaluate insomnia symptoms and severity [18]. The ISI is a seven-item self-report questionnaire that assesses the severity of problems with sleep onset, sleep maintenance, early morning awakening, sleep dissatisfaction, interference of daytime functioning by sleep difficulties, noticeability of sleep problems by others, and distress caused by sleep difficulties. The total score ranges from 0 to 28 [19].

The patients were divided into two groups. Group 1 had acute CSC, including those in the first episode, having spontaneous resolution of subretinal fluid within 3 months, and with no recurrence within 1 year. All these patients were monitored with OCT every 4 weeks for 1 year. Group 2 had persistent or recurrent CSC, including those in the first episode with persistent subretinal fluid (>3 m) and in the second or subsequent episode. The patients in Group 2 were prescribed half-dose PDT once the persistent or recurrent CSC was established.

### 2.3. Statistical Analysis

The data were analyzed with SPSS for Windows version 21.0 (SPSS, Chicago, IL, USA). The calculated values are presented as frequencies (proportions), means ± standard deviations, or medians (P25, P75). The Kolmogorov–Smirnov test was used to confirm the normality of the data. For the evaluation of the factors possibly associated with persistent or recurrent CSC, a standard two-step approach was followed: univariate and multivariate logistic regression analysis. The outcome of CSC was treated as a dichotomous variable (0 = acute CSC and 1 = persistent or recurrent CSC). In the univariate analysis, Student's *t*-test, Mann–Whitney–Wilcoxon test, chi-square test, and Fisher's exact test were appropriately implemented. A *p* value < 0.05 was considered statistically significant.

## 3. Results

In total, 138 patients were enrolled in the study. Of these, there were 20 (14.5%) with acute CSC and 118 (85.5%) with persistent or recurrent CSC. The latter included 46 with persistent CSC and 72 with recurrent CSC.  Table 1 shows the demographic and clinical data of the study sample and the results of the univariate and the multivariate analysis. Male sex, older age, and higher ISI score were independently associated with persistent or recurrent CSC.

The present case-control study showed that male sex, older age, and higher ISI score were independent risk factors for the persistent or recurrent CSC in a Chinese population.

## 4. Discussion

The present case-control study showed that male sex, older age, and higher ISI score were independent risk factors for the persistent or recurrent CSC in a Chinese population.

The reported male : female ratios in CSC patients ranged from 2.6 : 1 to 7 : 1 [7, 20, 21]. Chatziralli reported that male sex is an independent risk factor for the onset of CSC [8]. And our study confirmed that male sex is an independent risk factor for recurrent or persistent CSC. In addition, Hanumunthadu reported that women with CSC tended to have better outcomes than men [22]. These suggested that CSC might be associated with the regulation of male gender-specific steroid hormone. Testosterone is a vasoactive hormone that activates vasodilation [23]. The choroid is a highly vascularised tissue [24]. In CSC, choroidal thickening is associated with hydrostatic pressure increase and the hyperpermeability of the choroid, resulting in neurosensory retinal detachment [2]. Therefore, it is possible that testosterone is somehow related to the development and persistence of CSC by thickening the choroid. Haimovici reported one CSC case had elevated serum testosterone level near the time of presentation with acute CSC [16]. Nudleman reported nine cases developed CSC due to exogenous testosterone therapy [25]. Çiloğlu reported significantly elevated serum testosterone levels in CSC patients compared with age- and sex-matched controls [26]. Moreover, Forooghian and Moisseiev reported that antiandrogen treatment for CSC, using finasteride, was effective [27, 28]. Spironolactone and eplerenone, which had been studied for the treatment of CSC [29, 30], also had antiandrogen effects [31]. Although the underlying mechanism remains poorly understood, those male CSC patients may require careful follow-up or early PDT.

Consistent with previous studies [13, 20, 32], this study showed that age is an independent risk factor for persistent or recurrent CSC. It has been considered that overwhelming of the barrier function of the RPE contributed to the fluid leakage from the choroid to the subretinal space in CSC [2]. In aged human maculae, RPE cells increased in size and lost their regular hexagonal shape [33]. A study of aging monkeys observed mitochondrial elongation in the RPE cells within macular area, which was an indicator of increased metabolic stress [34]. In aged mice eyes, RPE cells decreased in number and undergo multinucleation because of failure of cytokinesis [35]. All these observations indicate that the repair capacity of the RPE may decrease with age, leading to persistent CSC [13], and that the RPE in aged eyes may be vulnerable to hydrostatic pressure increase and the hyperpermeability of the choroid, resulting in recurrent CSC. Our data suggest that early PDT should be considered for older patients with persistent CSC. And, all the CSC patients, even those young patients who had spontaneous resolution of subretinal fluid within 3 m, require regular and constant follow-up. With increasing age, the risk of recurrence may increase.

It has been reported that sleep disturbance is significantly more frequently observed in CSC patients than in matched control individuals [9, 12]. Sleep disturbances have been associated with increased activities of the hypothalamic-pituitary-adrenal axis and the autonomic sympathoadrenal system, which are characterized by altered secretion of cortisol and catecholamines hormones [36]. These two hormones are implicated in the pathophysiology of CSC [37]. Studies have also shown that CSC patients have higher levels of urine and plasma cortisol than control subjects and increased levels of plasma catecholamines [16, 38–40]. All these indicated that sleep disturbance is associated with the development of CSC. In the present study, we showed that high ISI score was independently associated with persistent or recurrent CSC, suggesting that sleep disturbance may also have a negative effect during the process of CSC. Therefore, the treatment of sleep disorders is strongly recommended for both treatment-naïve patients and post-PDT CSC patients with sleep disorder, to reduce the risk of persistent or recurrent CSC. Unlike sex and age, sleep disorder is the only risk factor for CSC that can be treated or controlled.

Haimovici reported that alcohol use was an independent risk factor for the onset of CSC [7]. Ethanol was oxidized into acetaldehyde (AcH) in the liver, and AcH can cause vasodilation [41]. And Kang reported that the thickness of choroid increased significantly shortly after ethanol consumption [42]. Therefore, it is hypothesized that ethanol consumption may have a negative effect to some extent in patients with CSC by thickening the choroid [42]. However, in this study, we failed to find the relationship between monthly ethanol consumption and the recurrence or persistence of CSC. Possible reasons may include that in this study, accurate data on the amount of each time and frequency of ethanol consumption were not considered, so the original data are insufficiently precise. Moreover, individual variations in the expression of AcH dehydrogenase, which converts AcH into harmless acetic acid, may also influence the effect of AcH on vasodilation, so the ethanol intake alone may be insufficient. Although the data in this study did not reach statistical significance, the biological plausibility of the vasodilation effects of alcohol worsening CSC is intriguing and should be studied.

Previous studies have compared CSC patients with normal controls to identify the risk factors for the development of CSC. However, we are more interested in the difference between patients with acute CSC and those with persistent or recurrent CSC. Therefore, our results should offer practical suggestions for the management of CSC patients. For patients considered to experience acute CSC (who are female, young, and enjoy good-quality sleep), a waiting strategy with regular follow-ups is reasonable, with PDT an optional therapy. However, for those deemed to suffer persistent or recurrent CSC (who are male, elderly, and/or have poor sleep quality), PDT is better applied early. The management of the risk factor by the treatment of sleep disorders should be recommended to the appropriate CSC patients. These risk factors could also be useful in designing future randomized studies of CSC, to limit any potential bias. We hope that the identification of these risk factors sheds some light on the mechanism of CSC.

The limitation of this study included the small sample and especially the small group of patients with acute CSC. Ozkaya reported that approximately 1/4 CSC patients experienced spontaneous resolution of subretinal fluid and without recurrence within 1 year [43]. However, in this study, the proportion was only 1/7 of patients. This may have been a response to selection bias because the majority of CSC patients who were experiencing their first episode and recovered quickly were not referred to or did not attend to our hospital (a tertiary hospital). Another limitation was that we did not analyze the corresponding multi-imaging information. Further studies that include more clinical information may tell us more.

## 5. Conclusion

This study has shown that male sex, age, and sleep disorder are risk factors for persistent or recurrent CSC in the natural history. Male patients, older patients, and those suffering poor sleep quality may require early PDT. Treatment for sleep disorder should be strongly recommended. All the CSC patients may require careful and periodic follow-up.

## Figures and Tables

**Table 1:** Demographic and clinical data and univariate and multivariate analysis of risk factors for persistent or recurrent central serous chorioretinopathy.

Exposure	Acute CSC patients (*n* = 20)	Patients with persistent or recurrent CSC (*n* = 118)	Univariate analysis		Multivariate analysis	
			cOR (95% CI)	*p*	aOR (95% CI)	*p*
Male sex	14 (70.0)	95 (80.5)	1.770 (0.614–5.106)	0.291	5.634 (1.023–31.024)	0.047
Age	41.00 ± 7.06	46.29 ± 8.84	1.086 (1.016–1.161)	0.015	1.137 (1.034–1.249)	0.008
Hypertension	1 (5.0)	26 (22.0)	5.370 (0.686–42.023)	0.109	2.140 (0.228–20.116)	0.506
Depression	4 (20.0)	24 (20.3)	1.021 (0.313–3.336)	0.972	1.474 (0.338–6.434)	0.606
Allergic diseases	4 (20.0)	23 (19.5)	0.968 (0.296–3.172)	0.958	3.670 (0.826–16.310)	0.088
Tobacco use	7 (35.0)	52 (44.1)	1.463 (0.545–3.930)	0.450	0.870 (0.227–3.332)	0.839
Life changes	3 (15.0)	17 (14.4)	0.954 (0.252–3.608)	0.944	0.603 (0.119–3.066)	0.542
Stress at work	11 (55.0)	47 (39.8)	0.542 (0.208–1.407)	0.208	0.520 (0.163–1.661)	0.270
Monthly amount of alcohol consumed (standard drink)	0.00 (0.00, 10.14, range 0.00–84.55)	3.38 (0.00, 33.81, range 0.00–169.07)	1.019 (0.995–1.043)	0.121	1.020 (0.993–1.048)	0.146
Monthly shift work hours	0.00 (0.00, 10.25, range 0–171)	0.00 (0.00, 45.75, range 0–360)	1.009 (0.995–1.023)	0.232	1.008 (0.992–1.025)	0.338
Sleep beginning time^*∗*^	−1 (−2, 0, range −3–1)	−1 (−2, 0, range −4–7)	1.115 (0.823–1.511)	0.483	0.966 (0.542–1.720)	0.906
Sleep hours per day	7.5 (7, 8 range 5–8)	7.0 (6, 8 range 2–11)	0.805 (0.584–1.110)	0.185	1.207 (0.677–2.154)	0.523
ISI score	1 (1, 4, range 0–10)	3 (1, 9, range 0–22)	1.174 (1.011–1.364)	0.035	1.298 (1.051–1.604)	0.015

aOR = accurate odds ratio; CI = confidence interval; cOR = crude odds ratio; CSC = central serous chorioretinopathy; ISI = Insomnia Severity Index; age is presented as mean ± standard deviation; other continuous variables are presented as median (P25, P75, range); all categorical variables are presented as number (%). ^*∗*^Sleep beginning time was reset: we defined 12 am as 0, 11 pm as −1, 1 am as 1, and so on. Thus, the sleep beginning time was rendered a continuous variable.

## Data Availability

The questionnaire data used to support the findings of this study are restricted by the Ethics Committee of Eye and Ear Nose Throat Hospital of Fudan University in order to protect patient privacy. Data are available from Qing Chang (qngchang@aliyun.com) for researchers who meet the criteria for access to confidential data.

## Abbreviations

AcH: Acetaldehyde
CSC: Central serous chorioretinopathy
FA: Fluorescein angiography
ISI: Insomnia Severity Index
OCT: Optical coherence tomography
PDT: Photodynamic therapy
RPE: Retinal pigment epithelium.
